# Enhanced Empathic Pain by Facial Feedback

**DOI:** 10.3390/brainsci14010005

**Published:** 2023-12-20

**Authors:** Seoyoung Lee, Yeonjoo Yoo, Heeyoung Moon, In-Seon Lee, Younbyoung Chae

**Affiliations:** 1 Acupuncture and Meridian Science Research Center, College of Korean Medicine, Kyung Hee University, 26 Kyungheedaero, Dongdaemun-gu, Seoul 02447, Republic of Korea; seoyoung.lee@khu.ac.kr (S.L.); alice98113@khu.ac.kr (Y.Y.); heeyoungmoon91@khu.ac.kr (H.M.); inseon.lee@khu.ac.kr (I.-S.L.); 2 Department of Behavioral Medicine, Faculty of Medicine, Institute of Basic Medical Sciences, University of Oslo, 0372 Oslo, Norway

**Keywords:** autonomic response, corrugator supercilii, empathic pain, facial feedback hypothesis

## Abstract

The facial feedback hypothesis states that feedback from cutaneous and muscular afferents affects our emotion. Based on the facial feedback hypothesis, the purpose of this study was to determine whether enhancing negative emotion by activating a facial muscle (corrugator supercilii) increases the intensity of cognitive and emotional components of empathic pain. We also assessed whether the muscle contraction changed the pupil size, which would indicate a higher level of arousal. Forty-eight individuals completed 40 muscular contraction and relaxation trials while looking at images of five male and five female patients with neutral and painful facial expressions, respectively. Participants were asked to rate (1) how much pain the patient was in, and (2) how unpleasant their own feelings were. We also examined their facial muscle activities and changes in pupil size. No significant differences in pain or unpleasantness ratings were detected for the neutral face between the two conditions; however, the pain and unpleasantness ratings for the painful face were considerably higher in the contraction than relaxation condition. The pupils were considerably larger in the contraction than relaxation condition for both the painful and neutral faces. Our findings indicate that, by strengthening the corrugator supercilii, facial feedback can affect both the cognitive evaluative and affective sharing aspects of empathic pain.

## 1. Introduction

Empathy is the phenomenon of being aware of and understanding how another person feels, without conflating one’s feelings with those of the other [1]. Cognitive, emotional, motivational, and behavioral processes interact and play a role in the multifaceted construct known as empathy. Many studies have been conducted on the cognitive and affective components of empathy [2]. The “cognitive component” of empathy is evaluating another person’s emotions, which relates to theory of the mind [3,4]. Perspective-taking and reading facial expressions are both cognitive evaluative aspects of empathy [5]. The term “emotional empathy” is used to describe the vicarious sharing of emotion; the emotional components engage a partial affective sharing of others’ affective states [6]. For instance, individuals vicariously experience the unpleasantness associated with the pain someone else is feeling. These two types of empathy should be considered simultaneously.

As facial emotional expressions are essential for human interaction, various studies have investigated their role in the comprehension of emotions [7]. The facial feedback theory of emotional efference was introduced in 1884; William James hypothesized that the expressive muscles contraction or relaxation may have a causal role in our experience of emotions [8,9]. According to this idea, emotional experience and facial feedback are related, and the facial efference modifies physiological and psychological responses to facial expressions [10]. In a previous study that compared valence ratings for pictures and text during smiling (contraction of the zygomaticus major muscles) and frowning (contraction of the corrugator supercilii muscles), participants had a more positive opinion of the text when smiling compared to when frowning [11]. Botulinum toxin treatment of the muscles in the upper face prevents facial expressions such as frowning, and reduces perceptions of negative emotions [7].

Facial feedback may also modify autonomic reactions to emotional stimuli. Facial feedback of arousing emotions such as fear, anger, and happiness, affects autonomic responses by increasing the sympathetic activation associated with emotion, as well as the intensity of the induced emotions. For example, the expression of facial efference generally increases the skin conductance response (SCR), an indicator of sympathetic arousal, while the deliberate blocking of facial expressions reduces the SCR [12,13]. A previous study evaluated the SCR, pupil size, and facial electromyogram (EMG) of the corrugator supercilii muscle as participants were imitating or passively observing angry facial expressions on a screen [14]. Actively imitating another’s facial expression of anger produced considerably greater responses for all three measures (SCR, pupil size, and EMG) than passive observation, suggesting that facial feedback enhances autonomic responses during an emotional experience. Empathic pain can be produced by considering another’s painful experience. However, as of yet no study has investigated whether facial feedback enhances the ability to empathetically evaluate and share another’s pain. Therefore, in this study, we tested whether actively frowning and, thus, contracting the corrugator supercilii muscles influences empathy for another person’s pain.

The aim of this study was to investigate if enhancing negative feeling by stimulating a face muscle (corrugator supercilii) increases the intensity of cognitive and emotional components of empathic pain, based on the facial feedback hypothesis. We hypothesized that the enhancement of negative affect by activating the corrugator supercilii would increase the intensity of cognitive and emotional components of empathic pain. We also investigated whether or not muscle contraction changes pupil size, which would indicate a higher level of autonomic arousal.

## 2. Methods

### 2.1. Participants

In total, 48 participants (26 females and 22 males) were recruited to this study. All participants were neurologically and physically healthy and had no major diseases. The participants were recruited through print and online advertisements. None of the participants wore glasses during the experiment. They all had normal or corrected vision. All subjects provided informed consent before the study, which was conducted according to the guidelines of the Human Subjects Committee and approved by the Institutional Review Board of Kyung Hee University, Seoul, Republic of Korea (approval number: KHSIRB-21-243).

### 2.2. Experimental Design and Procedures

In total, 20 images from the Delaware Pain Database [15] were used: 10 experimental images of a male (*n* = 5) or a female (*n* = 5) with painful facial expressions, and 10 control images of the same models with neutral facial expressions. With a focus on painful and neutral expressions, the Delaware Pain Database is a fully defined, varied collection of images that is accessible to the public and contains 240 distinct subjects’ specific painful expressions. All stimuli and associated norming data can be found online “https://osf.io/2x8r5 (accessed on 10 July 2021)”. Inadequacies in the size, homogeneity, characterization, and stimulus variability of facial expressions of pain were reduced by the database. To reduce racial ingroup bias or the other race effect, we solely used images of Asian people from the database in this study [16,17].

The facial images were centered on the screen, with the ears and neck removed. The experimental stimuli were displayed on a 51 cm monitor located approximately 80 cm from the participant’s eyes (maximum size of 23 × 29 cm). The participants were told to relax while seated in front of the monitor, and to keep their bodies still. Electromyographic electrodes were placed on the medial end of the corrugator supercilii and mastoid bone, and we calibrated the eye-tracking system to track pupil size and gaze.

To compare autonomic responses between facial expression stimuli, we recorded autonomic responses while the participants viewed neutral and painful facial expressions on the screen. A fixation cross was shown for 1 s in the middle of the screen at the start of each trial. Then, two non-facial cues were displayed for 3 s, in a random order. The participants were instructed to contract their corrugator muscles in response to cues with two yellow arrows (contraction cue), but not to cues with two yellow dots (relaxation cue). This process was falsely described to the participants as “a way to calibrate the movement of the eyebrows”. Facial expression images were displayed for 2 s after a 1s rest period.

**Figure 1:**
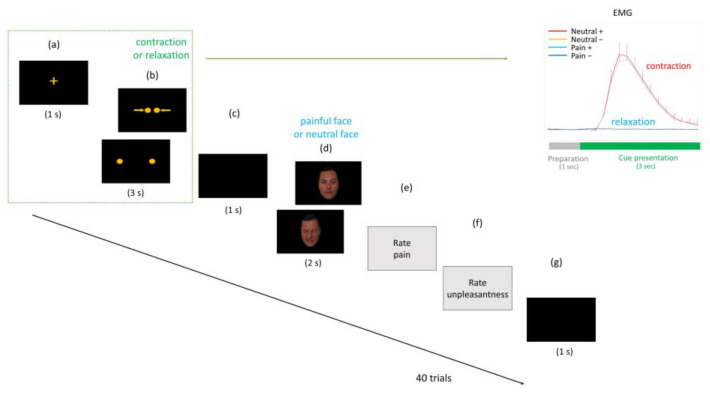
Overview of the experimental design. (**a**) Each trial started with a fixation point at the center of the screen, which was shown for 1 s. (**b**) One of two cues (contraction or relaxation of the corrugator muscle) was shown randomly; participants were asked to imitate this for 3 s. Contraction of the corrugator was represented by the two yellow arrows. (**c**) Participants were asked to relax for 1 s without making any facial movements (rest period). (**d**) One of the facial expression images (painful or neutral faces) was then presented randomly for 2 s. (**e**) After viewing the images, participants were asked to rate the model’s level of pain, as well as (**f**) the intensity of their own unpleasant feelings. (**g**) Each trial ended with a 1-s rest period. Electromyogram activities were measured during presentation of the corrugator muscle cues (contraction or relaxation). Enhanced corrugator muscle activities were observed after presentation of the cues associated with contraction (red and sky blue lines), but not after the cues associated with relaxation (blue and pink lines). The 40 trials per experiment all lasted ≥8 s (including the rating period).

The participants rated their unpleasant feelings and pain at the end of the trial (cognitive evaluative aspect of empathic pain, on a 6-point Likert scale: “Please rate the intensity of the pain of the person in the image” [0 = no pain at all, 5 = greatest possible pain]; affect sharing aspect of empathic pain: “Please rate the intensity of your unpleasant feelings while watching the person in the image” [0 = no unpleasant feelings at all, 5 = strongest possible unpleasant feelings]). The 40 trials all lasted more than 8 s (including the rating period, which was displayed without time limit) (Figure 1).

### 2.3. Facial Electromyogram and Pupil Size Measurements

EMG signals were recorded from the corrugator supercilii muscle using a ground electrode placed below the mastoid bone, and electrodes on the medial end of the muscle, according to standard facial EMG guidelines [18]. The PowerLab 8/30 instrument (ML870; AD Instruments, Bella Vista, Australia) was used. The EMG data were bandpass-filtered (1 kHz–0.3 Hz; 10 mV). Phasic facial EMG activity was defined as a change from the baseline activity, which was calculated for 1 s before the stimulus onset. The EMG data from each trial were retrieved every 0.2 s over a total of 4 s (1-s fixation cue and 3 s of non-facial cues).

The pupil size was measured during the test using a computerized eye-tracking system (iView X^TM^ RED; SensoMotoric Instruments, Teltow, Germany). The pupil size of each participant was calculated when the gaze fixated on each neutral or painful face for 2 s during the contraction or relaxation condition. The pupil size of each participant was measured by averaging the pupil size during neutral or painful face (2 s) between the contraction and relaxation condition. The BeGaze (SensoMotoric Instruments) eye-tracking program was used to analyze the data.

### 2.4. Data Analysis

Values are expressed as mean ± standard error. The EMG and pupil size responses were averaged within the same trials and stimuli. For the subjective ratings and pupil size, we conducted a 2 × 2 analysis of variance (ANOVA) of the pain and unpleasantness ratings with two within-subjects factors: facial expression (neutral or painful face) and facial feedback (contraction or relaxation). Statistical analyses were performed using the R statistical software package (ver.3.6.0; http://r-project.org) and Jamovi software (ver. 0.9; http://www.jamovi.org). A *p*-value < 0.05 was considered significant.

## 3. Results

### 3.1. Pain and Unpleasantness Rating

**Figure 2:**
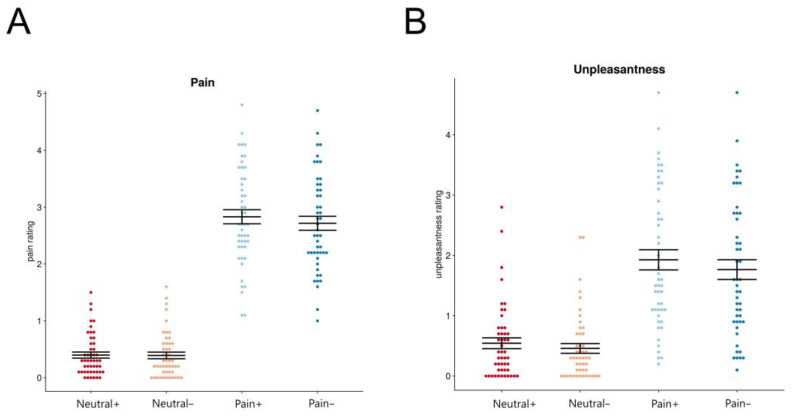
Pain and unpleasantness ratings. (**A**) Pain ratings for painful and neutral faces: comparison between the contraction and relaxation conditions. Pain ratings for the painful face were significantly higher under the contraction than relaxation condition, but no significant difference in pain ratings for the neutral face was observed between the contraction and relaxation conditions. (**B**) Unpleasantness ratings for the painful and neutral faces: comparison between the contraction and relation conditions. Unpleasantness ratings for the painful face were significantly higher under the contraction than relaxation condition, but no significant difference in unpleasantness ratings for the neutral face was observed between the contraction and relaxation conditions. “Pain+”: viewing painful faces after muscle contraction (sky blue), “Pain−”: viewing painful faces after muscle relaxation (blue), “Neutral+”: viewing neutral faces after muscle contraction (red), “Neutral−”: viewing neutral faces after muscle relaxation (pink).

The pain ratings revealed significant main effects of the facial expression analyzed with ANOVA (*F* = 331, *p* < 0.001) and facial feedback conditions (*F* = 4.61, *p* < 0.05), and a trend toward a significant interaction effect (*F* = 3.73, *p* = 0.056). Pain ratings in response to the painful faces were significantly higher under the contraction than relaxation condition (2.83 ± 0.13 vs. 2.72 ± 0.12, *t* = 2.899, *p* < 0.05), but there were no significant differences in pain ratings for the neutral face between the contraction and relaxation conditions (0.40 ± 0.05 vs. 0.39 ± 0.06, *t* = 0.151, *p* = 0.999) (Figure 2A).

The unpleasantness ratings revealed significant main effects of the facial expression analyzed with ANOVA (*F* = 55.90, *p* < 0.001) and facial feedback conditions (*F* = 15.35, *p* < 0.05). The interaction effect was not significant (*F* = 1.56, *p* = 0.215). Post hoc analysis revealed that the unpleasantness ratings for painful faces were significantly higher under the contraction than relaxation condition (1.93 ± 0.17 vs. 1.76 ± 0.17, *t* = 3.67, *p* < 0.01), but no significant differences were observed in the unpleasantness ratings for neutral faces between the contraction and relaxation conditions (0.54 ± 0.09 vs. 0.46 ± 0.08, *t* = 1.88, *p* = 0.244) (Figure 2B).

### 3.2. Pupil Size Changes

There was a significant main effect for the facial feedback condition (*F* = 26.771, *p* < 0.001), but not for the facial expression condition (*F* = 0.039, *p* = 0.843); the interaction effect was also non-significant (*F* = 0.020, *p* = 0.888).

**Figure 3:**
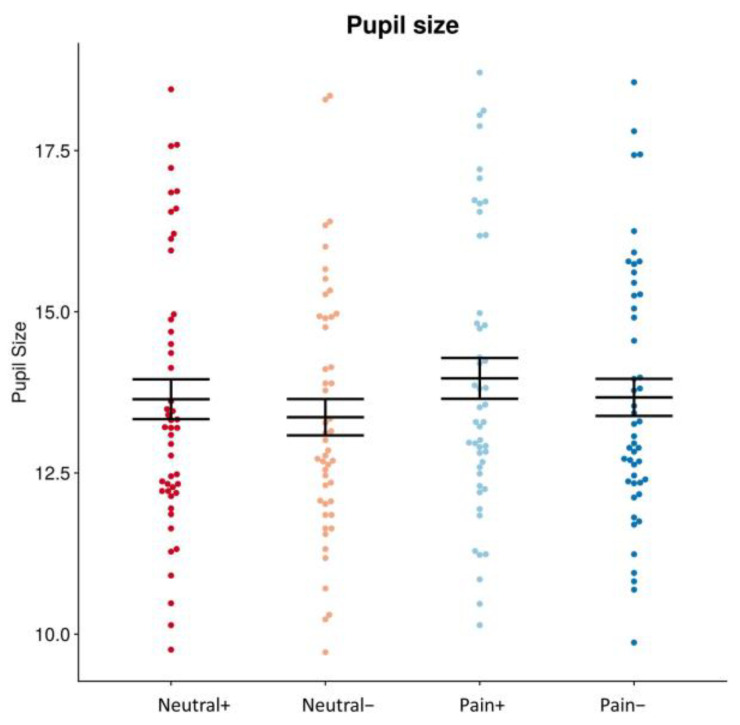
Pupil size while viewing painful and neutral faces after the muscle contraction and relaxation conditions. Pupil size was significantly larger while viewing painful faces after the contraction than relaxation condition. Pupil size while viewing neutral faces was significantly larger after the contraction than relaxation condition. “Pain+”: viewing painful faces after muscle contraction (sky blue), “Pain−”: viewing painful faces after muscle relaxation (blue), “Neutral+”: viewing neutral faces after muscle contraction (red), “Neutral−”: viewing neutral faces after muscle relaxation (pink).

Post hoc analysis revealed that the pupil size while viewing painful faces was significantly larger under the contraction than relaxation condition (13.97 ± 0.32 and 13.67 ± 0.29, *t* = 3.778, *p* < 0.01). Furthermore, the pupil size while viewing neutral faces was significantly larger under the contraction than relaxation condition (13.64 ± 0.31 vs. 13.37 ± 0.28, *t* = 3.540, *p* < 0.01) (Figure 3).

## 4. Discussion

We investigated the role of facial feedback in the cognitive and affective components of empathic pain. Based on the facial feedback theory, we assessed how people perceive others’ facial expressions. As expected, the participants experienced more unpleasant feelings and perceived another’s painful face as more painful when the corrugator supercilii muscle was contracted than when their face was in a neutral state. Pupil size was significantly greater under the contraction than relaxation condition in response to the painful and neutral faces.

In the current study, facial feedback had a significant effect on empathy for another person with a painful face. These results are similar to a previous study in which imitation of another person’s facial expressions induced a similar emotion in the observer, leading to emotional contagion and empathic reactions [19]. The effect of facial feedback on empathy for others in pain was reported in a study that explored whether individual differences in empathy modulated the sensitivity of facial feedback. Participants in the high emotional empathy group were more likely to rate a film as funny when they were asked to produce a happy expression compared to the low emotional empathy group [20]. We suggest that the facial feedback hypothesis can help explain empathy for others in pain through cognitive and affective mechanisms.

Among the various facial muscles involved in facial expressions, we analyzed the activity of the corrugator supercilii muscle. In studies based on the Facial Action Coding System, “narrowed eyes with furrowed brows and wrinkled nose” were identified as major characteristics of painful faces [21,22]. In a previous study, the activity of the corrugator supercilii muscle was positively correlated with the empathic concern score, which is a measure of the empathic response. The correlation between the level of empathic concern and EMG activity of the corrugator supercilii muscle suggests that the latter reflects the empathic response induced by emotional mimicry [23]. Thus, we expected enhanced corrugator supercilii contraction to promote empathy for another’s painful experiences and emotional responses (effect of facial feedback on empathic pain).

We also recorded pupil size while testing the effects of facial feedback on empathic pain. Pupil size is regulated exclusively by the autonomic nervous system and reflects the level of arousal [24]. The pupil size is significantly greater in response to emotional than neutral stimuli, regardless of valence [25]. In this study, regardless of the facial expressions of others, pupil size increased when viewing faces after corrugator supercilii contraction compared to the neutral condition, indicating that the muscle contraction itself may have significantly increased sympathetic activity and arousal. Further investigations should evaluate how increased sympathetic activity and arousal influence cognitive and affective components of empathic pain.

Empathic pain is particularly important in the clinical setting given that most patients visit the hospital to alleviate pain, and that empathy can be beneficial during the treatment process. A meta-analysis revealed a modest benefit of empathy and the provision of positive messages to patients in terms of pain management [26]. Another study reported a positive correlation between patient-rated physician empathy levels and patient satisfaction with pain consultations, which underscores the importance of empathy in the clinical setting [27]. Future research is necessary to determine how active or passive mimicry of a patient’s facial expressions by clinicians affects doctor-patient interactions, as well as the clinical outcomes of pain and other medical conditions.

To our knowledge, this is the first study to investigate the effect of facial feedback (frowning) on perceptions of painful and neutral faces of others. It has been suggested that pupil dilation is a broad measure of a person’s level of arousal and alertness. In this study, the observed rise in sympathetic activity, reflected in changes in pupil size, could be potentially associated with the heightened experience of empathic pain triggered by facial feedback. Our results may provide preliminary ideas of the underlying behavioral and autonomic mechanisms of facial feedback and empathic pain.

This study had some limitations. First, simply viewing the painful and neutral faces may not have evoked significant empathic pain in some participants. Future studies should present stimuli depicting pain in others in greater detail, such as videos and both facial and bodily expressions of pain. Second, the direct effect of facial feedback on empathic pain merits further investigation given that empathy can be affected by many factors, and that muscle contraction itself may alter emotional and arousal states.

## 5. Conclusions

In conclusion, we explored the effect of facial feedback on empathic pain. Strengthening the corrugator supercilii can impact both the cognitive evaluative and affective sharing aspects of empathic pain. The increased sympathetic activations measured by changes in pupil size might be linked to the increased empathetic pain by facial feedback. We suggest that contraction of the corrugator supercilii muscle enhances the facial feedback effect, thereby increasing the intensity of cognitive and emotional reactions to pain in others.

## Data Availability

The datasets used and/or analyzed during the current study available from the corresponding author on reasonable request. The data are not publicly available due to privacy and ethical restrictions.

## References

[1] Decety J., Lamm C. (2006). Human empathy through the lens of social neuroscience. Sci. World J..

[2] Cuff B., Brown S., Taylor L., Howat D. (2016). Empathy: A review of the concept. Emot. Rev..

[3] Blair R.J. (2005). Responding to the emotions of others: Dissociating forms of empathy through the study of typical and psychiatric populations. Conscious. Cogn..

[4] Reniers R.L., Corcoran R., Drake R., Shryane N.M., Vollm B.A. (2011). The QCAE: A Questionnaire of Cognitive and Affective Empathy. J. Pers. Assess..

[5] Clark M., Robertson M., Young S. (2018). “I feel your pain”: A critical review of organizational research on empathy. J. Organ. Behav..

[6] Zhang H.B., Ou H., Meng D.H., Lu Q., Zhang L., Lu X., Yin Z.F., He C., Shen Y. (2021). Impaired Cognitive Empathy in Outpatients with Chronic Musculoskeletal Pain: A Cross-Sectional Study. Neural Plast..

[7] Alam M., Barrett K.C., Hodapp R.M., Arndt K.A. (2008). Botulinum toxin and the facial feedback hypothesis: Can looking better make you feel happier?. J. Am. Acad. Dermatol..

[8] Behera S., Rangaiah B. (2016). Identificaton of emotional maturity among traditional dancers: As a function of dance style, gender and residency. Int. J. Indian Psychol..

[9] McIntosh D.N. (1996). Facial feedback hypotheses: Evidence, implications, and directions. Motiv. Emot..

[10] Adelmann P.K., Zajonc R.B. (1989). Facial efference and the experience of emotion. Annu. Rev. Psychol..

[11] Meeten F., Ivak P., Dash S., Knwoles S., Duka T., Scott R., Kaiser J., Davey G. (2015). The effect of facial expressions on the evaluation of ambiguous statements. J. Exp. Psychol..

[12] Lanzetta J.T., Cartwright-Smith J., Kleck R.E. (1976). Effects of nonverbal dissimulation on emotional experience and autonomic arousal. J. Pers. Soc. Psychol..

[13] Tourangeau R., Ellsworth P.C. (1979). The role of facial response in the experience of emotion. J. Pers. Soc. Psychol..

[14] Lee I.S., Yoon S.S., Lee S.H., Lee H., Park H.J., Wallraven C., Chae Y. (2013). An amplification of feedback from facial muscles strengthened sympathetic activations to emotional facial cues. Auton. Neurosci..

[15] Mende-Siedlecki P., Qu-Lee J., Lin J., Drain A., Goharzad A. (2020). The Delaware Pain Database: A set of painful expressions and corresponding norming data. Pain Rep..

[16] Huo T., Shamay-Tsoory S., Han S. (2023). Creative mindset reduces racial ingroup bias in empathic neural responses. Cereb. Cortex.

[17] Stelter M., Schweinberger S.R. (2023). Understanding the mechanisms underlying the other-‘race’ effect: An attempt at integrating different perspectives. Br. J. Psychol..

[18] Fridlund A.J., Cacioppo J.T. (1986). Guidelines for human electromyographic research. Psychophysiology.

[19] Hsee C., Hatfield E., Carlson J., Chemtob C. (1990). The effect of power on susceptibility to emotional contagion. Cogn. Emot..

[20] Andreasson P., Dimberg U. (2008). Emotional empathy and facial feedback. J. Nonverbal Behav..

[21] Kunz M., Prkachin K., Solomon P.E., Lautenbacher S. (2021). Faces of clinical pain: Inter-individual facial activity patterns in shoulder pain patients. Eur. J. Pain.

[22] Meister E., Horn-Hofmann C., Kunz M., Krumhuber E.G., Lautenbacher S. (2021). Decoding of facial expressions of pain in avatars: Does sex matter?. Scand. J. Pain.

[23] Sun Y.B., Wang Y.Z., Wang J.Y., Luo F. (2015). Emotional mimicry signals pain empathy as evidenced by facial electromyography. Sci. Rep..

[24] Pfeifer M.A., Cook D., Brodsky J., Tice D., Parrish D., Reenan A., Halter J.B., Porte D. (1982). Quantitative evaluation of sympathetic and parasympathetic control of iris function. Diabetes Care.

[25] Partala T., Surakka V. (2003). Pupil size variation as an indication of affective processing. Int. J. Hum. Comput. Stud..

[26] Howick J., Moscrop A., Mebius A., Fanshawe T.R., Lewith G., Bishop F.L., Mistiaen P., Roberts N.W., Dieninyte E., Hu X.Y. (2018). Effects of empathic and positive communication in healthcare consultations: A systematic review and meta-analysis. J. R. Soc. Med..

[27] Walsh S., O’Neill A., Hannigan A., Harmon D. (2019). Patient-rated physician empathy and patient satisfaction during pain clinic consultations. Ir. J. Med. Sci..

